# Gleanings from Discussions at the Recent Meeting of the I. H. A.

**Published:** 1889-09

**Authors:** 


### HOMŒOPATHIC PHYSICIAN,

A MONTHLY JOURNAL OF

HOMŒOPATHIC MATERIA MEDICA AND CLINICAL MEDICINE.

If our school ever give up the strict inductive method of Hahnemann, we are lost, and deserve only to be mentioned as a caricature in the history of medicine."—CONSTANTINE HERING.

Vol. IX. SEPTEMBER, 1889. No. 9.

### GLEANINGS FROM DISCUSSIONS UPON PAPERS READ AT THE RECENT MEETING OF THE INTERNATIONAL HAHNEMANNIAN ASSOCIATION.

### I.—What is Contagion?

Dr. J. T. Kent's paper, entitled "The Healing Principle," being under discussion, the question as to "What is Contagion?" arose; or, better, the question might be put, "When are people susceptible to contagion?" We give some of the views expressed:

Dr. Long—I have for sixteen years taken the stand that diseases are not contagious. It requires such a body as this to understand such a remark, and I have been censured over and over again for trying to explain that fact.

I have slept with diphtheria. It was the ill-wind that blew me good sixteen years ago. When I attempted to establish a practice, I started on an epidemic of diphtheria. I have slept with it and been with it for hours, and I have never used a preventive except my natural health, and I have never had a sign of sore throat. I think Dr. Kent brings out one fact—that is, if you are not in perfect health, you are sick and susceptible to the various diseases that are prevalent. Again, I have been with small-pox and was never vaccinated since a baby. It is generally understood by the laity that physicians use something—it is got from the practices of the old school. I remember fifteen years ago, visiting with an allopathic physician, who actually smoked at the bedside of his patient. So that if you clear up the question, " What is contagion?" you are relieving a great wound to the physician.

Dr. Biegler—I don't know that I can add my thoughts on anything valuable, but when the Doctor brings up the question of contagion I cannot help but state the stand that I take. I have always maintained that the contagion is the disease; if the disease is cured the contagion is done away with, instead of beating the air, creating a smudge, calling out the military, and making a noise to kill the microbes. If the patients in Florida were cured, and the patients in Italy and France, and the cholera and yellow fever patients—if they were cured, the contagion would very soon be ended.

In regard to the susceptibility of a patient, to the individual there is good ground for understanding the manner in which the contagion is received. Dr. Kent's paper makes that so plain as to render it unnecessary to go into it, but, like Dr. Long, I have been exposed to diphtheria for thirty years, and had the membrane coughed into my lips; it has even gone into my eyes, and I have not yet succumbed to that disease. I have no theory framed to answer an experience of this kind; it does not entertain the least theory. I am not susceptible, and when I am asked this, as I have been asked within the past three or four days, I simply say I am not a subject for it. That is why I have escaped. I wish to make that point here. I believe the best thing that can be done to subdue contagion in disease is to cure the patient, and not as scientific men do now, let the patients die and try and kill the microbes. The sooner the patient is cured the sooner the disease is cured.

Dr. Reed—It is needless to add to Dr. Kent's paper, but as confirmatory of what he has said I might state that I was in charge of a small-pox hospital in 1862–63 in the army. I never had been vaccinated and never will be. I am very much like "Buffalo Bill" when he went into Paris; the officers said that all of his men would have to be vaccinated, but he replied: " If we are not allowed to enter as we are we will not enter Paris at all, and you will not have the privilege of seeing our show." I have also two other instances to state. I remember one man that had the small-pox in the ambulance with me, and I never was susceptible to it, and never had a skin disease in my life.

This susceptibility that Dr. Kent has brought out—we are not sick when we are not susceptible to prevailing miasma. There is a lady in our city who cannot pass by a house where the painters are at work without having lead colic; she will invariably go by on the opposite side of the street. Another lady of my acquaintance, *cannot think* of eating a strawberry without producing an eruption upon her body—her little girl can eat all she pleases without any evil result—and it can be explained in no other way than that given by Dr. Kent.

Dr. Ballard—I was only thinking what a valuable paper this was; I feel very much obliged to Dr. Kent for the paper; I never was satisfied with the idea of Rhus poisoning that the case was cured. The patient needed Rhus before. Dr. Kent has furnished the explanation—because the Rhus was indicated.

In another case, I remember Anacardium cured very quickly. This paper explains a great deal, and although it is truly Hahnemann's teaching to say a patient is not sick if there are no symptoms, yet I think we will have to modify that to such a degree as Dr. Kent has shown. The patient is sick, because capable of being made sick in certain ways, if we can discover that fact. I have slept with diphtheria and never had a touch of it in my life, but I have not seen much small-pox.

Dr. H. C. Allen—I wish every physician in the land could explain as thoroughly, as simply, and as easily as Dr. Kent has to-night the cure of Rhus poisoning by Rhus-tox; how it would open their eyes! If you should speak to the majority of homœopaths about curing Rhus poisoning by Rhus-tox, they would say, "you are isopathic, you are practicing isopathy." The patient is simply calling for Rhus before poisoning; he was extremely susceptible to that action.

I have been poisoned with Rhus when a boy without going within fifteen or twenty feet of Rhus, and several years ago I learned the secret of taking along Rhus$^{2c}$, and if I was exposed I took a dose of Rhus and had no further trouble. As Dr. Ballard says, I have slept with diphtheria, and treated small-pox in a malignant form, and never had anything of the kind. There is no danger of a healthy person becoming sick unless there is a susceptibility to disease, and the cure of that susceptibility renders him capable of throwing off anything of the kind, and Hahnemann distinctly points that out in his volume of *Chronic Diseases.* The majority of our young men have not the *Chronic Diseases*, and possibly if they had it they would not read it. I have been told Hahnemann is an old fogy, and that we now know a great many more things about the science of medicine than Hahnemann ever knew about "curing disease," and we are not to be taught by him. I am positively certain of the truth of the statement made by Dr. Weldon, President of the Ohio State College, in which he said that every homœopathic college in the country was not doing its duty when they did not have a special chair devoted to the *Organon* of Hahnemann. That is what we need to-day—teachings of this kind to our students—then we would not require a College of Homœopathics.

Dr. Butler—People with idiosyncrasies that render them liable to the action of drugs or disease make most excellent provers, and they will develop symptoms which no other prover will, therefore the *Materia Medicas* that are about to come out are going to omit the most valuable symptoms—*determinative symptoms.*

Dr. Emory—I have been very much interested in Dr. Kent's paper; and, as expressed by Dr. Ballard, it has cleared up Rhus poisoning being antidoted by a high potency of Rhus; that was brought up at a meeting of the Hahnemann Club not long ago, and we were not agreed as to why a high potency of Rhus will apparently cure Rhus poisoning. Like Dr. Ballard, I am not through thinking about it yet.

Dr. Dillingham—I have been interested in Dr. Kent's paper very much. I think the first explanation of the action of Rhus is very remarkable, and if it be true it occurs to me that he has done away with the theory of isopathy. If his explanation of Rhus is correct, the same will be true of all the remedies introduced under the head of "*nosodes,*" because some are susceptible to Rhus and others are not—it is the same in contagious diseases but after another kind, and I would like to ask Dr. Kent whether his theory does not do away with the theory of "isopathy?"

Dr. Kent—Mr. President, I had intended to refer to this in the summing up, as I have the last word. I will say now, as we all know, that this miserable bugbear, "isopathy," has been staring us in the face, it worried me into suggesting a solution of the difficulty, as I have done for you in this paper, and it remains with you to say as to whether I have furnished the solution or not to the bugbear "isopathy;" many things have been presented in isopathic cures that I have been unable to explain away.

The question has been asked "Would you give a high potency of Morphia to antidote a hypodermic of Morphia?"—I have also put into its place the susceptibility of other poisonous substances—I have made an explanation of the principle, and it remains with you to follow it up and we will communicate hereafter. This is only the beginning; I can offer this view that is expressive of what has been revolving in my mind, and we will develop something after awhile.

Let us, then, not refuse this subject, but meet it like men, like philosophers, like physicians. Are these cures Homœopathy under the disguise of isopathy? I believe they are homœopathic. Wherever they occur I believe they are permanent —cures are only effected under the homœopathic law; but, on the other hand, reliefs are apparent, they are short-lived. We have antipathic relief, such as we find sometimes produced by a repetition of high potencies, that will produce antipathic cure, but only in a very tough disposition. You wonder why the patient is as susceptible to the curative remedy as he is to the disease, that he is unable to resist the cure—a mere smell of the bottle—and he is unable to resist the cure, he cätches it (as it were); it is by contagion, and he is cured.

We have the same demon for cure as we have for making sick, only when a person gets too much he is made sick, but when cured he gets just little enough.

When he is not susceptible enough to the medicine, what can you produce but antipathy, and your relief is a deception, a fraud, and a snare.

Dr. Baylies (New York)—Will the single medicine, when exhibited in high potency, damage the case? We all admit the injurious complications with disease resulting from the crude drugs; and as homœopathicians asserting the powerful action of high potencies administered for proving, we must either believe that the diseased body excludes their action when non-homœopathic, or that they also, when thus administered, complicate the case and embarrass the cure. If the principle explaining the so-called isopathy just announced by Dr. Kent be accepted—that the supposed antidote appears so to act because it was homœopathic to the state preceding the poisoning—it would follow by analogy that the non-homœopathic, absolutely dissimilar medicine, especially if administered in high potency, would not damage the case or interfere with the action of the simillimum.

Dr. Campbell—I would like an explanation as to whether that same principle can be applied by Dr. Kent when he cures Quinine cases by the high potency of Quinine; would the same principles apply?

Dr. Kent—Do you mean immediately or long after? It applies long after because he is no longer suffering from the effects of Quinine, but from the chronic affection left by the dynamis, and not the crude drug-effect.

Dr. H. C. Allen—It is the same thing of tobacco; it acts in the same way—the high potency is the best antidote for tobacco and the 2C potency of Quinine the best antidote for Quinine, and it is permanent; that is my experience, *provided the drug be let alone.*

Dr. Sawyer—High potencies of Coffea will do the same.

Dr. H. C. Allen—I know a mother of an allopathic physician that carries a bottle of Coffea $^{2c}$, and whenever she has a cup of coffee she also takes a dose of Coffea $^{2c}$.

Dr. Kent—Because she is susceptible to Coffea. It is the susceptibility you aim at with your high potencies. We will take a case of poisoning by Morphia, where the patient is not susceptible, but received it by accident in large quantity. The question has been asked—and rightly—would you expect to give a high potency to one who is dying from the effects of Morphia? I have been asked that question—I don't think you would. The explanation is that it is not that kind of a case.

Dr. Butler—It does not seem possible that bad cases of tobacco poisoning will be cured by high potencies of Tobacco. How many times have you used Sabadilla, and ought you not to have used it if you did not do so, and persons poisoned with Quinine ought to have Pulsatilla, or is it that the Quinine symptoms manifest themselves so strongly that the high potency (of Quinine) will have to be given, and, similarly, for other drugs at other times.

Dr. Kent—There is another portion in that paper—I made the statement that frequent repetition of the poison increases or brings about that susceptibility, and though the individual may be only partially sensitive to it, he becomes poisoned with it, and afterward susceptible to the merest inhalation of it, and over sensitive after having once been poisoned with it. The individual who has been in the habit of taking Quinine and becomes sensitive to it—it is the chronic effect—and the old sensitiveness is cured by the repetition of the Quinine in high potency—and it is the same with Coffea as Quinine.

Dr. Ballard—I think there are a great many of our thoughts we have not known how to explain. We all know that any one of us may step into the house, say a few words to some lady, and nothing is felt and nothing wrong about it; we might say these same words to another lady and she would swoon away. In the first case, there is no homœopathicity at all, and in the second case there was a condition of that woman which made this rumor homœopathic, because of the relationship between the person spoken of and the lady. The same thing exists in the actions of medicines in all cases. We find a person who has been drugged, and we give a remedy homœopathic to the condition; we simply remove the plug and let that poison float away. It had found a suitable soil, but had it not found a soil in which it could take root it would not have been felt at all. Now, it is the same thing in regard to contagion, it may be found on all hands. Hahnemann says our medicines cure by producing a disease stronger than the one which the patient is suffering from, but I think Dr. Kent's explanation of the case better than that remark itself; and I have always contended that a dose of high potency of any remedy could not produce a disease stronger than the one from which the patient was suffering. The patient who is on the sick-bed with small-pox: the remedy simply acts homœopathically to the condition, as the virus or contagion of small-pox does to the susceptibility of that person taking it. And we have to-night simply opened a vial and let loose the odor which has been corked for a long time, the substance of which we now see in the bottle but we don't know how to get it out; but next year we can express ourselves as to it. I might say, in order to emphasize my remarks in regard to the homœopathicity of the words spoken to the lady—An Irishman who was sent to break the sad tidings to the wife of the sudden death of her husband (Mr. Kelly)—he being a great friend could do it a great deal better than any one else. On rapping, Mrs. Kelly came to the door. "You are the lady of the house, I suppose?" "I am Mrs. Kelly," she answered. "Your are a liar, for the corpse is coming around the corner."

### II.—Germs.

As closely related to the subject of contagion, the question of the part played by the so-called "germs" is one of the greatest interest. Every since these germs were discovered and the practice known as "Listerism," based upon it, has been in vogue, there has been no one subject which has so constantly occupied the attention of the "old school." All this Dr. Bell shows in his paper, and moreover he clearly demonstrates that the germ theory, with the practice based upon it, has not given the surgeons the good results claimed for it.

Dr. Stow—I think this Association is vastly indebted to Dr. Bell for this remarkable paper. There is one thing in connection with the paper that appeared to me at first to suggest something—I cannot give the words exactly—but the Doctor, if I understand it correctly, said in speaking of the causes of the formation of microbes, that we are ignorant of them.

I venture this suggestion that it will not be found to be the most reasonable view to take of it. Since that in every solution of continuity of animal structure you have severed nerve filaments which convey to the parts that amount of nerve force requisite to maintain them in a condition of health, and hence until there is some restoration of the tracks through which this force is carried, you will have breaking down of tissue and production of microbes, and so forth, and that carries us back also to this alternative theory of Hahnemann, *that it is a derangement of the vital forces*—whatever that may be—which gives us symptoms of disease and produces disease. To reach that vital force is to reach the matter in hand and (to) cure the case.

Dr. J. B. G. Custis—This paper has given me a great deal of pleasure. I like to see members exhaust subjects in the way Dr. Bell has this. I do not know if I can add anything to it, but only just show how far they are carrying on this discussion in Washington.

I have made inquiries of Professor Schmitt there in the employ of the Government studying this subject, and he is bending every energy toward the isolation of bacteria and the reproduction in the healthy organism of the disease from which they are supposed to have been isolated, and all he would say was that they had reproduced the "pear blight"—the disease in the pear tree—and reproduced it in all its peculiar characteristics in the pear tree, but would not claim *that anything* had ever been reproduced with its characteristics in the healthy man; he believed it had been done in *animals*, but could not prove it. I think it is important for us to have put ourselves on record both as investigators of this subject and of having firm convictions after having investigated it.

Dr. Ballard—I wish to sustain the paper by a little experience. It always seemed absurd to me that we have traveling through our vessels a menagerie which we must kill or destroy before we can exterminate the disease.

A year ago a patient of mine had amputation performed at the upper third of the left thigh because of necrosis of the femur, and there was also enlargement and chronic suppuration of one of the inguinal glands and a sinus connected with the opening of the thigh. That wound was carefully dressed and bandaged every day for nearly six weeks, but there was no healing. They tried to heal this inguinal gland by pressure, and finally one of our noted professors of surgery expressed the belief that it would have to be dissected out. All the while the wound was dressed on the "Listerian plan."

I gave the remedies which appeared to be indicated for the different symptoms as they came along and would control them, but the wound would not heal, and these symptoms, or something in addition to them, would be continually cropping up.

I finally said to the surgeon, Now, I don't believe this nonsense any longer; this young man is a patient of mine, and Listerism cannot save him, my remedies are prevented by Carbolic acid from doing their work, and so I will exterminate everything from that wound (of such a nature).

The pus was highly odoriferous, and every little while, if the pressure was removed from the inguinal gland it would bulge up, because of the pressure somewhere else from the pus not being freely discharged. I gave the indicated remedy, which was "Sulphur," and I can safely say that within ten days that stump was healed, which had been discharging for six weeks, and did not break out again, either.

So, in addition to the vital force which may resist this menagerie, I believe the next best antiseptic is the *simillimum*.

Dr. Schmitt—In support of Dr. Ballard, I want to tell you of another menagerie, a case of erysipelas, caused by a wound on the skin of the tibia. The erysipelas went down into the superficial tissue and made a very long wound over an area of large size. The patient was dresser to our hospital, and that fact alone will show you that there was no Carbolic acid or other disinfectant there but a simple dressing of Pencoline to prevent irritation. The remedies with which I healed the foot were Silicea, followed by Hepar-sulph. and the whole case was well in five weeks.

In the allopathic hospitals they would have cut off that foot.

### III.—Arnica or Calendula?

Dr. Alice B. Campbell read a paper entitled "In Praise of Calendula," which caused quite a discussion upon the indications for the use of Arnica and Calendula.

(Dr. Campbell's paper will be found in full in our July issue.)

Dr. Bell—Dr. Campbell has made the point that torn tissue indicates Calendula.

Dr. H. C. Allen—There is a very broad distinction between the use of Calendula as a universal application; here it is used on its indications perfectly correct—well done in praise of Calendula.

Dr. Dillingham—I want to say one word—in some cases it is confusing to know what to use—we have three remedies for wounds—Arnica, Calendula, and Hypericum. In Dr. Campbell's case Arnica was the remedy, at least, I can hardly see where she could give Calendula and Arnica, but the patient took two remedies; one remedy must have done the whole thing. A properly-dressed wound and the indicated remedy would have done much.

Dr. Campbell—That question has occurred to me, but it seemed to me the boy was so shocked that Arnica appealed to the mental condition, and that was why I gave it the first night and in two days afterward the pain had gone. There was restless sleep, desire to sleep but could not. I gave a dose of Belladonna, then left him on the Calendula to heal the external wound. I had a little experience with that same young man with a pistol shot. He got shot in his hand with a pistol, and his mother was up all night bathing him with Arnica, and next day there was a line of inflammation extending up the arm, which I took to be the poisonous effects of Arnica, but it did not bias me if I had the indications of Arnica. I thought the mangled condition of the wound would be helped by Calendula, and at the hospital they commended my proceeding; they continued it themselves, and I do not think they are accustomed to do so. The wound healed beautifully and rapidly under the Calendula.

Dr. Nash—I would like to know if Calendula is the specific for lacerated wounds and Arnica for shock, and when we have both conditions present which are we going to use?

Dr. Dillingham—If I remember, my impressions are correct in regard to the use of Calendula—it should be given for clean-cut wounds, and Arnica given for bruises—and it was indicated in this case; it was the remedy.

Dr. Schmitt—Several years ago I had some experience with lacerated wounds, and one week a fellow would come in with a crushed finger—the flesh crushed on the bone, and I generally cleaned the wound and put Calendula tincture on it undiluted to stop the bleeding—it generally does so at once—and then wrapped it up and left the wound for several days.

Dr. H. C. Allen—Did you give nothing internally?

Dr. Schmitt—No, I don't now give anything internally.

Dr. Long—I have had some experience and I find, like in everything else, there is no exception in medicine, and that Calendula does not cure in every case of lacerated wounds. I would like to ask Dr. Schmitt if he ever had a case that was not lacerated.

Dr. Schmitt—Some of them suppurated, and if I noticed any bad smell the bandage was taken off, and I generally gave any remedy which was indicated and kept the wounds open to see them.

Dr. Campbell—Would you then stop the Calendula?

Dr. Schmitt—Yes, I stopped the Calendula.

Dr. Campbell—Is there a law regulating that fact of mangled or bruised surfaces? I only apply Arnica if the surfaces are cut.

Dr. J. B. G. Custis—I am afraid to use Arnica where there is no abrasion. I have seen several bad cases, one of which proved fatal, resulting from the use of Arnica.

Dr. Bell—What kind of Arnica?

Dr. J. B. G. Custis—I was going to speak of that, and I instruct my patients never to use Arnica unless greatly diluted. I have used Arnica exceedingly diluted so that you could hardly detect the odor in the water, where there has been a great deal of contusion in addition to the laceration, but the Calendula in preference to that if the surface is simply cut. There has been some Hamamelis in Washington which has been dangerous to use. One lady hurt her eye, and upon the advice of her friends applied Hamamelis and the whole side of her face became swollen—it also occurred in another case.

These articles should be prescribed in a homœopathic drug-store. Their purity is very necessary in order to form a judgment as to their virtue.

Dr. Stow—Ought not this view to be taken: I would wish to distinguish between the Arnica and Calendula in all cases where the flesh has been bruised without breaking it, where the life quality has been knocked out of the tissue, and where ecchymosis follows rapidly, *Arnica* is the remedy, but if you come to a wound that is torn as with a buzz-saw, and it is mangled—only such parts of the wound, as the ragged portions, are likely to become destitute of vitality—in that case give Calendula. If the wound is large the torn parts should be chipped off so as to make the wound clean. Those distinctions ought to be borne in mind in the treatment of wounds. Then in regard to the pathogenesis of Arnica—what is that condition calling for Arnica? In typhoid fever it is where there is rapid loss of vitality, presented very largely at times in bleeding sordes and those peculiar ecchymosed spots upon the skin indicating rapid breaking down of tissue—here Arnica is the remedy. There is a two-fold trouble here—when a ball strikes the flesh it bruises it, compresses it and makes a hole. In such a case it is embarrassing to know which to use, the Arnica or the Calendula, and it depends upon the amount of laceration following the ball. If the wound closes there is a danger of accumulation of matter unless there is drainage.

Dr. Dillingham—There is another question I want to mention. To-day surgery is a complicated affair. The surgery of the future is to be the simplest possible surgery. In this case reported, what carried the patient through, and what did the cure? Because as Hahnemannians we are bound to give the indicated remedy, and treat the wound in the simplest possible way. In one of the hospitals of England they use nothing but dry dressings. We find here a wound being done up by a mechanic, by wrapping it up simply in the blood. We want to throw off in the treatment of wounds everything that is not absolutely necessary, otherwise we are getting complicated as Hahnemannians.

Dr. H. C. Allen—There is another point, and that is this: We prove, and we use our remedies in the potentized form; why should we come down and begin at once with the tinctures in the treatment of wounds? If we use Arnica take the 30x in water, or some other way. We get just as good results if you get the right preparation of Arnica—not that made from the leaves, because then you get insect poison similiar to the Cantharidis which is invariably found in the arnica leaves, and it is impure because of this fact. Our allopathic friends throw it away; they are afraid of using it. There is no necessity of using either tincture of Arnica or Calendula, use the potenized preparation of it. Dr. Campbell has not cleared up the distinction why she used Arnica *and* Calendula. If Arnica was to be used, that alone should be used, if Calendula, that alone; then we gain the point and our friends of the A. I. H. will say there is alternation of remedies in the I. H. A.

Dr. Nash—I think it was Dr. Fore who brought forth Calendula as a remedy for wounds, and told of the peculiar virtues of this remedy, and in order to convince them made a deep cut in the fleshy portion of his hand, dressed it in Calendula, and the next day appeared before the company with his hand perfectly healed. It is said Calendula is a remedy for those wounds that suppurate profusely. I have seen suppuration rapidly subside under the use of it in a burn which covered the back and hips of a child who fell into a pail of hot water. The suppuration was profuse and I used Calendula, with the effect of rapidly healing up the wound. But I would like to inquire whether the fact is established that Calendula is a remedy for profusely suppurating injuries by internal use. Dr. Allen claims it is a specific remedy which may be used just as well in the potency internally as in the tincture. Is it an excellent remedy where suppuration is profuse?

Dr. Biegler—I would not take your time in giving indications for Calendula, Arnica, and Hamamelis, but I wish to have the fact impressed that Dr. Allen has just brought up—that those remedies act *better in the highly diluted form than in the tincture.* I never, of late, have used any of those preparations except in the very highly diluted form, and I have had perfectly satisfactory success in using Arnica by being careful to obtain the homœopathic tincture—I have frequently got erysipelas from the other—by dropping ten or twelve drops into half a pint of water, which is just as effectual. So with Calendula and Hamamelis. Now as to the use of the potentized form, we obtain here better results. I have used it on burns. A solution of the *CM* of Canthar., locally in water, with perfect satisfaction. Take a little of the preparation *CM* dissolved in a few spoonfuls of water, then fill an eight-ounce bottle and use locally.

Dr. W. H. Reed—I had a case of a lady who had rhagades of the nipples, a distinctly marked case of Sulphur. I had heard that the topical application of Sulphur would be advisable to use, and laid a cloth soaked with the *CM* potency upon the nipples and got great relief, but afterward cured with a dose of Graphites.

Dr. Custis—In the peeling off of the epithelium of new-born children it has been customary with me to use Argentum-nitricum $^{2c}$, a few pellets in water, and the nurses claimed there was something magical about it.

Is there not some difference between the effect of Hamamelis tincture or dilution and the potency of it in its power to check hemorrhage—will a hemorrhage from the lungs be checked as rapidly by Hamamelis when not in high potency, as when in the dilution or tincture? It seems to me there is a special therapeutic force in the potencies which is not in the tinctures, while there is drug action in the tincture which we want to get rid of in the potencies.

Dr. Bell—I think we must struggle against routine practice, and would say in connection with these cases, I have tried in every case to give the indicated remedy where required.

Arnica when there is pain in the operation—local pain—*Staphisagria* where there is pain in other parts—perhaps remote—after an operation, after lithotomy or amputation of the breasts if there is colic or strangury.

Dr. H. C. Allen—What remedy does Dr. Bell use in the terrible vomiting after abdominal operations?

Dr. Bell—There are two kinds: One after Ether, and the other due to peritonitis, and the latter is best treated by opening the abdomen and washing out the matter, but in the other case there is no remedy except as indicated by the symptoms. In other words, only rely upon our principles as ordinarily practiced.

### IV.—Magnesia-phosphoricum; a Proving of the CM Potency by Olfaction.

Dr. Taft reported: I took three powerful whiffs in each nostril of the CM potency of Mag-phos. and waited two days; on the third day I forgot it, but at dinner, at one o'clock, I had to leave the table and lie down. I was so very cold and chilly—chills up and down the back—headache; throat very sore; the subjective symptoms on right side, and objective on left; constant desire to swallow, which symptom remained for several weeks. It seemed to me like a hot corn-husk lodged in the throat, and I had to swallow constantly for weeks. Pain in the back of head, worse in the right frontal region; all the symptoms relieved by heat and covering. I had a bag of hot water at my feet and another at my head. I was shivering all over—teeth chattering—spasmodic yawning; it seemed as though it would dislocate my jaws, and tears rolled down the cheeks from it.

I was doubtful whether all this came from the remedy or not; but if it came from handling the CM, I don't know whether I dare go on with the five potencies Dr. Allen sent me.

Dr. Allen—Those who have never attempted to prove a drug by olfaction had better begin with Camphor and see how wonderfully it acts. I read a proving by olfaction on beginning the practice of Homœopathy, and that was enough to satisfy me that the whole thing was a humbug, instead of proving it for myself as I should have done.

Dr. Taft—I am doubtful whether the symptoms came from that.

Dr. H.C. Allen—I have had the same symptoms occur in several provers, and know they came from it. I have another report made by Dr. Campbell—a proving of Mag-phos. made by one of her patients.

Three doses of the 2c were taken in this proving. Enlargement of the joints has extended over both hands; I gave one dose of Lycopodium and they subsided in about a week, but returned, but are subsiding now.

The symptoms produced in Dr. Taft and this prover—the chills running characteristically down the spine (*vs.* Gels.)—and afterward run up and down the spine: like Gels. This has been characteristic of all the provers and all the potencies; but the higher potencies have produced it more promptly and the action was more prolonged. A doubtful allopathic physician was scoffing at our potencies, who did not think there was anything in them, said he would investigate Homœopathy if we could show him how these small doses acted. We were shrewd enough to demonstrate the action of the dose by getting him to begin the provings. The result is that to-day he is investigating Homœopathy and becoming a pretty lively homœopath.

Dr. H. P. Holmes reported a case cured by Mag.-phos.

A man who had been washing sheep had sciatica and could not lie down. All the sleep he got was in a chair, and hot applications to the right sciatic nerve was all the relief he could get. He could not lie down on account of pain. Mag.-phos.$^{30x}$, cured him without much trouble. (It is not always right side.)

This is a remedy that will rival many of our Polychrests, as Calc.-carb., Bell., Rhus, Veratrum, and many others. All we want is a little further verification, and this we should get from the members of this Association.

I gave one of my provers a vial of the 30x and 2C, and asked her to prove what she could and use it in her dispensary, having an admirable opportunity to investigate the action of this drug. Her report was that there was hardly a dispensary clinic for the last ten months in which she had not been required to dispense Mag.-phos., and never failed to obtain prompt relief, and she almost entirely relied upon Mag.-phos. Often, in all these cases, she never had a symptom of the remedy, and could not tell a single symptom on which to prescribe.

Dr. Campbell—I have a report of a case of dysmenorrhœa that lasted for some time, in which at each menstrual period a membrane was discharged varying in size from one to two inches long. She came to me for this trouble. The principal symptoms were: she was taken after the flow began with severe pains in the abdomen low down, which were relieved by lying curled up in bed with a hot-water bag on the abdomen. The pains would last perhaps for a day—dull, aching pains—and next day or the day after a membrane would be passed. She was in very good health with this exception. I gave her after one of her menstrual periods Mag.-phos.$^{cm}$, one dose dry. The next menstruation was easier somewhat, but not much. I think I repeated the Mag.-phos. in water, but have not the record. She got it, I

think, in water for two days, night and morning, and the next menstrual period was painless, though she passed the membrane as before. After that the menses were perfectly painless. She had always stayed in bed before without any relief. She kept well for six or eight months; then she got her feet wet just before one of her periods, and she required and got a dose of the same remedy (which might have been Pulsatilla). It relieved her, and she has had no trouble since.

Dr. Bell—The patient, a lady in middle life, with nervous temperament, a patient of Dr. Lippe's. The time Dr. Wesselhœft saw her, a year or more ago, and for neuralgic headache gave her a dose of Mag.-phos. She was so much relieved by this that he gave her to take home with her a vial of medicated pellets to take, if the case should require it. Dr. Kent knows the rest.

Dr. Kent—This lady came into my office one day with a most violent cough—very spasmodic—her face red as a blaze from coughing. She could hardly speak long enough to tell her other symptoms, because her whole time was taken up in telling of her cough. I found she had been in Boston, and had brought home a vial filled with pellets. I asked her how many doses of them she had taken. She did not know, but thought the medicine had given her so much relief that she should go on taking it. I wrote to Boston, asking if Mag.-phos. had been given, because the pain was relieved by pressure and heat, and I knew several were investigating Mag.-phos. at the time, and my suspicions rested upon Mag.-phos. She kept on coughing, and coughing, until I thought her head would come off, and the time came when I must either make a change in treatment or lose my patient. So I had to antidote the medicine. The peculiarity of the cough led me to give Lachesis, which stopped the cough at once, but she had coughed almost incessantly for three or four weeks.

Dr. Allen—Did you use any other antidote as a remedy?

Dr. Kent—I have had some trouble in antidoting these cases. I had a patient with very severe shooting neuralgic pains during the menstrual period, the pains were in the stomach and lasted the first day or two. The pain commenced in the back and came directly around and centered in the pit of the stomach. It was relieved by heat and pressure. I gave a dose of the 10M Mag.-phos., and she had no more pain. She came to me a year ago, when leaving the city, and asked for a couple of the powders in case the severe pains should return. I gave them to her as she suspected the pain was coming on, and she took them (50M) in five or six doses a few hours apart. I had over three months' fighting in antidoting those doses. I forget the remedies given, and I believe it to have been the result of Mag.-phos. A marked symptom was developed a month after taking the powders, sufficient to drive her home—a tenderness of the dorsal spine for four to six inches in extent, which lasted three months, then passed away. In several cases Mag.-phos. acted a long time by reproducing its wave of symptoms. I believe it to be a very long and deeply-acting medicine.

Dr. Nash—You counteracted the cough with Lachesis; by what symptoms?

Dr. Kent—I cannot recall the details.

Dr. Biegler—A retching cough?

Dr. Kent—In a measure, choking-retching; worse in a warm room, better in the open air.

Dr. Kimball—Dr. Kent wrote that the face was so red that she seemed as if she would choke to death, the cough was so uncontrollable. I think it was aggravated in a warm room and at night on lying down.

Dr. Kent—Some of the head symptoms were aggravated in a warm room.

Dr. Ballard—Amelioration from pressure is not found in Lachesis, *i. e.*, from hard pressure.

Dr. Kent—You find amelioration from pressure, and aggravation from hard pressure.

Dr. Reed—I have an interesting clinical case. A lady sent for me about eight P. M. to go to see her. She was suffering from great pain. In the meantime I had to go to the College, and they were about ready to send for another doctor. She was complaining of extreme tenesmus and tormina, from a constant desire to pass water and go to stool. Every time this pain would come on she would rise up in bed and bend forward, and the only relief obtained was by hot water. I put a little of B. and T., 2C, of Mag.-phos. in a glass of water, and gave a dose every fifteen minutes—the third dose cured the pain. She had also a cough day and night—one of Dr. Kent's own patients—and I perceived no benefit. She had also the red face in connection with this, and I perceived no benefit from Mag.-phos. in this case of the cough, but complete relief of the tenesmus and tormina. I said this is a Phosphorus cough, and gave her Phosphorus, going away with the assurance that all would be right by the next morning. On going back next morning, I found that the cough had kept up, notwithstanding the pain was gone—there was aggravagation of the cough. She had coughed over two weeks. I then gave a dose of Sulphur, and then went home, and came again next day, and found the same greeting. Then, remembering the fact that she had been one of Dr. Kent's patients, I recognized the failure. I then took out a bottle of Fincke's CM of Phosphorus, and in five minutes' time I said, your cough is better. She answered, "Doctor, I could have told you that five minutes ago." And she had no cough from that time, and has not had any since. Well, now, in confirmation of Dr. Campbell's case, I have had three cases of this excessive pain at the catamenial periods, characterized by sharp, cutting pains. A colored girl—a servant—she was as coarse as she could be—and every time the pains came on she would throw herself across the edge of the table. I gave her Colocynth$^{2c}$, then CM, but she got no benefit. Next time she had Mag.-phos. I had none of B. and T., and gave her 6x. At the next period there was some benefit, and no pain after the third period.

Another patient had the same experience, and she was completely cured by taking Mag.-phos.$^{6}$. Another was cured by 2C at the second period. All these cases were relieved by heat, and all characterized by bending over, and by pressing on the abdomen. They were cutting, lancinating pains.

Dr. Campbell—But no exfoliation of membrane?

Dr. Reed—I cannot say. I gave the remedy just before menstruation.

Dr. Campbell—I usually get better results just after. In my case the flow was quite profuse, bright red, and perfectly regular, because she would always stay in bed that morning. She would be quite sure that the flow would come on either in the night or early in the morning.

Dr. Nash—Any hysterical symptoms before or during the menses?

Dr. Campbell—No.

Dr. Hawley—Did the discharged membrane come afterward, or before?

Dr. Campbell—The membrane comes either the second or third day, but not during the pains.

Dr. H. C. Allen—Perhaps I can help you out of this; one of my provers developed that symptom. She had never had a menstrual pain in her life. She was always regular; but she has had menstrual colic ever since taking Mag.-phos., and has menstruated six to nine days too soon. Her usual time between the periods was twenty-eight days from the time it ceases until it begins again. There was an intensely sore, bruised feeling all through the abdomen, which continued for two days. At this time she had not taken any of the medicine for a month, but during her time of proving it she had taken a dose night and morning for two or three days—then stopped. The menstrual pains were somewhat peculiar, in that they were ameliorated by the flow—like Lachesis—resembling Zinc, and feeling better during the menstrual period. The characteristics of the menstrual pains Dr. Reed has described very brilliantly.

I have had three cases of dysmenorrhœa cured, after running months or years, by this remedy, Mag.-phos. Contrary to the usual belief, the higher preparations of this remedy clinically in practice, give very much more prompt and better results than the 6x, and in this, as in all others, they are infinitely more effective than the crude drug; and many of our men use it almost exclusively in dysmenorrhœa for symptoms of this character, and they give it for everything without reference to aggravation, amelioration, or anything else. The three best antidotes are Gelsemium, Belladonna, and Lachesis. I have had two cases antidoted in provers, because I did not think the 1M would produce any serious result, though the result was anything but pleasant for them.

Dr. Hitchcock—This is a most interesting subject, and I think we want to hear more. I move that this Bureau adjourn.

Dr. Ballard—We can have more time to-morrow, and as there is only one more paper, we ought to finish up this evening.

Dr. Hitchcock—I withdraw my motion for the present.

Dr. Ballard—This nasal symptom of the alternating stuffiness and free discharge from the nostrils, will be recognized, perhaps, under another remedy, though not exactly with those symptoms. The only remedy I have ever found—that is, the symptoms of the discharge from the nose in a gush, and I have had a numner of cases this past winter—is Badiaga. The discharge is almost exclusively confined to the left side, and it will come in gushes at times—she would have to use a large towel, it would come in such a gush. There was not the stiff condition of the nose in the meantime.

Dr. Nash—Does the nasal symptom appear in the proving?

Dr. H. C. Allen—Yes; it appears in the proving.

### V.—Sanicula.

Dr. Wm. Jefferson Guernsey having reported a paper, giving some experience with this new remedy, Dr. J. V. Allen then reported his experience.

Dr. J. V. Allen—I have had considerable experience with

Sanicula, and a great many similar cases to that of Dr. Guernsey, in which Sanicula was indicated in summer complaint. But it is especially to the eye symptoms of Sanicula I wish to refer. It is marked photophobia, without much inflammation—so marked that the patient cannot stand the light of day. He must close the eyes continually, and with this there is an awful discharge of thick yellowish and greenish matter, which excoriates the cheek or any part of the face which it touches. All of the cases —which were of long standing, and had failed to be relieved by the old-school physicians—were cured in a very short time by Sanicula. The photophobia was the first symptom to disappear.

Dr. Biegler—Was there no nasal affection?

Dr. J. V. Allen—In one case of a child, the discharge was greenish, and the nostrils and lips were excoriated, but that entirely disappeared under the action of the remedy very quickly.

### VI.—Melilotus Alba.

Dr. H. C. Allen—I have a few mental symptoms to which I wish to call the attention of the Association. A few years ago we made a proving of "Melilotus alba," and since that I have been paying great attention to its mental symptoms, having made four brilliant cures, which were generally of a very severe type. In three or four of the cases the papers had been made out to send the patients to the asylum, and Melilotus completely restored them. I want to call your attention to a remark Hahnemann made in regard to Veratrum album, in which he believes it capable of working many cures of insanity. Dr. Bowen says he is in the habit of prescribing it for all cases of insanity to reduce the hyperæmic condition of the brain. He has given Melilotus for that condition alone, thinking that as soon as he could reduce it he would then prescribe for the other symptoms; but Melilotus cured up the entire mental symptoms in the case. I have verified these following symptoms so often that I think they can be relied upon: Great mental confusion; unable to fix the mind on any subject; extremely suspicious; thinks an adversary is on his track seeking to arrest him; capacity for business entirely gone; memory and judgment impaired, constantly making mistakes as to what ought to be done, thought there was something supernatural in his always waking before three A. M. and not sleeping again; this was the first symptom that marked the onset of the attack. He was attacked with insomnia; would sleep pretty well between twelve P. M.

and three A. M,, but after that no more sleep. None next day, but a little in the evening, then after twelve P. M. a few hours, and waking promptly at three A. M. After a dose of Melilotus the insomnia disappeared and never returned.

The following symptoms presented themselves in another case: A comprehension that personal disaster had overtaken him, that he was going to the almshouse, could not be prevailed on to eat anything except refuse; would not speak except in monosyllables, locking the doors, fastening the windows, watching sideways to see if the officer was coming after him, was positive he was not at home; did not know his own house, but recognized the members of his family; thought he had been brought to the house at the cemetery and prepared to be buried next morning; great nervous and mental prostration. I tried to encourage him well against this prostration, but it was no use, the mental prostration was too complete.

I call the attention of the members to these particular symptoms of Melilotus because there are other remedies that may be more useful in the treatment of insanity and mental symptoms.

Dr. Nash—I had considerable experience with Melilotus, and one characteristic symptom is this excessive redness of the face, which always, in my experience, goes along with these mental troubles, and often precedes nose bleeding, which is apt to occur in these cases.

### VII.—Transverse Presentation; A Case with Some Remarks.

[This excellent paper, by Dr. C. W. Butler, was published in our July issue; we now add a part of the discussion which followed its reading.]

Dr. Kent—I will tell you something like Dr. Butler's. It was one of the cases of a midwife, experienced, well educated, and of thirty years' practice. I had seen a number of her cases and considered her highly accomplished; but she occasionally sent for me to share the responsibility—and this one was a tedious case. She had diagnosed a breech presentation, and she sent for me with a note, saying she expected a three days' job on hand, and wanted me to come and assure the family that though it should be three days it would come out all right. I went to the house, made an examination, and found a breech presentation. I admit it was rather hastily done, but I confirmed her diagnosis. The dilitation of the os was between a quarter and half a dollar, and I went through with the assurance to the family she requested me to make with all conscientiousness, and did not think anything more about the case. The next morning she called at my office; the patient had had a good many pains ; they were irregular and spasmodic ; she was a Pulsatilla patient and I paid more attention to the case by taking symptoms and seeing what remedy would help her through, and she said, Why didn't you tell me I had fooled you. I made a mistake in that diagnosis. I am an old fool to practice midwifery for thirty years and not know a head presentation. You knew it was a head presentation, and I was an old fool. That child was born head first. Now did the Pulsatilla do it? It was born in a few hours after I had left the house. I had only stopped a few minutes, and remarked "it may be a tedious case."

Dr. Nash—I was very much pleased with the graphic description of Dr. Butler's case. It seemed as though I could almost see the devil raised. But, while we are talking about the power of Pulsatilla, it seems to me that while I have no doubt this has been the case in many instances, and we have no doubt reports to that effect from those who ought to know—it seems to me that it is possible, this is not the only remedy capable of performing that kind of business, and we should seek to find out those indications which lead us to give Pulsatilla. I believe that Caulophyllum, when understood, may accomplish the same results; and while we have so many remedies they should make it their particular business to prove Caulophyllum and those remedies which have an action upon the generative organs of women, so that we shall know better what we can do with them.

Dr. Butler—It is not at all uncommon with us, without remedies, for the child to change positions at the last moment, if the waters are not yet broken ; it is easier for the child at the time to change. In my case the waters had broken half an hour before and the irregular contractions of the uterus had taken place. It seemed a very peculiar case, and when I gave a dose of Pulsatilla I had no idea of any results. The only parallel I have found is one Dunham gives. It is difficult to tell whether he takes version or evolution. The only case I know is where Valpool saw a shoulder presentation turned by Pulsatilla.

Dr. J. V. Allen—We know that the natural labor is the head, and if it is unnatural it is amenable to treatment, and I think that the medicine indicated homœopathically will relieve that which is unnatural and bring it back to the natural one. And, in my experience, any of the remedies indicated by the symptoms will relieve. One case was that of Dr. Guernsey's; he was sick, I went to the house; the lady had been in labor several hours, and it was a very cold morning. I noticed she was up every two or three minutes running around the room, with terrible pains in her limbs. I found no dilitation of the os, and I asked her why she did so. She said she usually felt better when in motion. I gave her Rhus-tox., one dose of the CM, and sat down to get warm, and she went to lie down and said she felt a good deal better and did not feel like getting up and walking around. The os was now dilated about the size of a dollar, and the labor was over in fifteen minutes.

Dr. Schmitt—I had a case almost similar to Dr. Butler's. I was called to see a case attended by a midwife also very experienced. She did not like me because of being a homœopath. I came in and asked her what was the matter. She said, "Examine yourself." I did so, and found a shoulder presentation, but did not say so, and I pulled down a hand to make sure. Then I said: "We have a shoulder presentation," and she agreed. The os was dilated slightly. I could not make version. The woman had pains in the abdomen. It was a Pulsatilla case, and I gave a dose of Puls.$^{2c}$. About ten minutes after she was fast asleep—no pains, a sure sign the Pulsatilla was acting. We went into the other room while she slept for an hour. I went home, saying everything would be all right when she awoke. I was called in the morning about eight o'clock, and when I reached the house the child was born. She said it was born by the breech.

Dr. Butler—The shoulder presenting, the breech was carried up toward the upper part of the fundus uteri, and the first action of the muscular fibres was upon the breech, which carried it around that way.

Dr. Schmitt—There was no mistake about the presentation, and if the remedy acts it often puts the patient to sleep. She slept from three o'clock to seven o'clock in the morning, and, after a few pains the child was delivered. The version, therefore, must have taken place during her sleep. With regard to what Dr. Nash said I will remind you of a case I mentioned in our meeting at Syracuse where Sepia$^{2c}$ restored the child.

Dr. Long—I would like to report a failure along this line. I wish to preface my remarks by saying it was due to the fact that the patient did not receive constitutional treatment prior to the labor, which is the most important part of obstetrics. Last November, a year ago, I had a fellow doctor drop in to take tea with me. I had been called at nine o'clock the previous night to a case of labor, primipara, and diagnosed it as a breech presentation. I thought I had elicited the whole history of the case, and was warranted in giving Puls.$^{1m}$. My patient was a very patient woman. She had known me for twelve or fifteen years. She did not call me during the night, as I had requested her to do unless she was relieved, but spent the night in walking around the room. In the morning I thought I was justified in giving Lycopodium. The os was sufficiently dilated for me to attempt version, as it seemed impossible for the breech to advance. I continued Lycopodium throughout the day, and spoiled the doctor's supper in the evening by asking him to go with me in this case. Remedies apparently were useless. I introduced my right hand and brought down the feet, and with his assistance, manipulated the external walls of the abdomen and delivered the child. I was frightened because the child hadn't a particle of skin from the knees down, and I feared I had done it with the use of my fingers. Here is the history of that case in a few words: This young girl had married a farmer, and they had to struggle for a living. During the summer months, while in the field picking peas, a large snake jumped at her and she was found unconscious in the field. Now, this woman certainly required constitutional treatment from August to November, when the child was born. Two or three physicians saw the baby. It was literally covered with sores. The father is tuberculous—two of the family died with phthisis. The child, from the effects of the summer's heat and the overheating of the mother, has been nothing but one mass of scabs from the head to the feet. To-day she has apparently recovered all her vitality; she has good skin, has teeth, and her feet, which were deformed, are become natural and normal, and I believe she has the making of a strong, healthy child. I believe all the conditions—the proper position and the whole hygienic treatment— should be attended to prior to the time of labor. That is the time to administer remedies. Pulsatilla, Nux vomica, Chamomilla, Lycopodium failed in this case.

### VIII.—Mastitis, Its Treatment, with a Repertory.

In our July issue we published this excellent paper prepared by Dr. Wm. Jefferson Guernsey. We now add to it the experience of many others as given in their remarks upon Dr. Guernsey's paper.

Dr. H. C. Allen—Does Dr. Guernsey puncture threatening abscesses?

Dr. Guernsey—I do not. Sometimes an abscess will point in a certain direction, and perhaps in a few days' time, under the influence of medicine, it will point in some other part, fully an inch away from the first place—and the reason for not puncturing the breast is that I believe had I punctured the breast, I would have done so in the place I expected to at the first (*i. e.*, where it first pointed), and the fact that it did not break there would have shown me that I had made a mistake should I have done so.

Dr. H. C. Allen—That is just where nature will do her own work and do it best, and where Graphites is given in a case in which frequent punctures have been made, it is not then required. It is not the thing to lance.

Dr. Emory—In confirmation of Dr. Sawyer's remarks, I might state that I had a case, a few years ago, of a lady who had had two children, and with both of them she suffered tortures from an abscess of the left breast, so much so that she dreaded the terrible knifing more than labor. She had never had homœopathic treatment. I assured her she would not have any gathered breasts under homœopathic treatment. There were indications for other remedies during pregnancy, and I did not use Graphites until after labor, when the same old pains began as formerly, accompanied by swelling and hardness in the cicatrices, but Graphites$^{15m}$ removed all the difficulty. I have never had to lance a breast.

Dr. Campbell—I think I would like to tell of a single case of mine. I followed upon another physician officiating at the birth and attended to the case. There was a very extensive erysipelatous inflammation involving the whole of the breast. I cured the case, but was summoned to attend the other breast. I had never had such an occurrence before, and treated that in the same way for a little while. It was in an advanced stage of inflammation when I first saw it. "When did this come on?" I asked. She said, "I had been meddling with it and would not have mentioned it if you had not spoken." I did not understand the circumstances of the case, but finally they came out. She had had injections of Carbolic acid per vaginum—I had inquired about that before and told her to discontinue them. After the first breast healed, she had commenced the injections again, and the abscess of the other breast was, I believe, the result of using this Carbolic acid. They both suppurated, but, as Dr. Guernsey said, "it pointed in one place, and broke in another." Has any one else had the same experience?

Dr. Baylies—I have had the same experience, and have used

Graphites under the same circumstances for a series of years, and in the same person.

Dr. Campbell—I mean metastasis following vaginal injections. Am I wrong in attributing that effect to such a cause? It seemed very dark to me.

### IX.—The Care of the Breasts, read by Dr. J. B. G. Custis.

Every nurse has some special ointment or salve which is a sure means of preparing the breasts for lactation, and for preventing them from getting sore! The treatment of the breast is often a very difficult one, and with the best of care we are often disappointed. But this much may be safely said, that this local treatment does not do much good. Our readers will peruse the following discussion with much interest:

Dr. Biegler—This is an important paper in so far as it presents points of consideration to the members of this Association, which involve the question of local application, which he recommends such as a decoction of tea, to previously harden the nipples; the application of Glycerine, and the naming of certain principal remedies for certain conditions and for diseased states. I hope the members will take this paper in hand.

Dr. Guernsey—It is a question whether the nipple requires to be hardened; or rather, I think it should be softened. And, as far as any local means are concerned, I don't know why in this, or any other case, our patients should hold to their fancies, or that we should deviate from what we think is right. If anything is used (as a local application), take the remedy and dissolve it in water, which is very simple and much the best way. For tenderness of the breast, "Sulphur" is the remedy, and for cracking of the breast (around the base of the nipple), Graphites. Dissolve the remedies in water with four parts of Alcohol, and apply after nursing.

Dr. Sawyer—I have had some experience in these cases. I have never yet seen a case of cracked nipples, sore nipples, and abscess of the breasts in the patient, free from miasm or other psora sycosis, scrofula, or other kind of taint, and the best results are obtained from treating the patient and not the nipple. Treat the patient every time. I permit them sometimes to make a cake of beeswax and soften it to the shape of the breasts, which only keeps the patient warm and allows her to do no harm. But the indicated remedy will do the work every time.

Dr. Nash—Dr. Guernsey says it is a question whether they ought to be softened or hardened. The patient wants them toughened; so I refer them to the tanner if they want them tanned. That, as Dr. Sawyer has just said, of late years I have tried these things and there's no objection to using glycerine, or egg, or beeswax to get it merely painted on the patient while we cure them constitutionally. But I have simply used a solution of the potency of some remedy the same as is given internally, and my success is much better.

Dr. Guernsey—We have no remedy which will produce such excessive soreness as *Apis mellifica*, and, to my mind, Dr. Sawyer's application may have benefited the nipple as much as the medicine did internally.

Dr. H. C. Allen—Dr. Sawyer will find out that occasionally the patient is so extremely sensitive to the action of Apis that she cannot stand the action of beeswax.

Dr. Sawyer—I shall not prescribe it again.

Dr. Bell—One of the great attractions of this Society is that the members are all ready to repent and reform. I think Dr. Campbell and Dr. Custis are going to reform. The application of tea is wrong; it is the same old story of green tea to the eyes. If the patient is psoric, tea will do no good. The patient should not be allowed to use improper means; and it should be our whole duty, and our success would be better, to follow the straight and narrow path and have nothing used externally except water or oil, just to occupy their minds while the remedy acts internally. Nothing externally can possibly be of benefit.

Dr. Schmitt—I have a holy terror of those old grannies with their stuff on the nipples. I tell them I am going to cure the nipples with medicine, but they don't believe it, though I make them believe it ultimately. Sulphur, Sepia, and Lycopodium are indicated in different cases.

Dr. Biegler—Although it is not in my province to discuss this paper, I cannot let the opportunity go by without expressing my objection to the use of glycerine as well as tea for external application. I think I have seen very serious results follow its application to the mucous surfaces.

Dr. Custis—I want to set this on record in the treatment of these cases. I think they take the brandy for the moral effect. Life is too short to go into a long argument with everybody, and if you can find something that will not do any harm: if you can give them a little license without a particle of injury to the patient, I don't think that there is any objection. I have never seen any harm from using glycerine, though some people's skin cannot stand glycerine. I have generally looked upon it as harmless, but if it is wrong I will give it up. The main point is that we get the expression of members on their exact method of practice in these cases.

Dr. Emory—I think one of the objections to these local applications is thus stated, as I once heard my late partner express it in reference to poultices in the formation of abscesses, pneumonia, etc., for which the old school use them. Years ago, when he was younger than now, he occasionally yielded to the wishes of his patients in this respect, and when he came back the next day the patient said, "Doctor what a grand thing that poultice was," and he knew that the change was not due to the poultice but to the remedy.

Dr. Custis—I want to refer to some criticism in regard to mentioning remedies for each of the conditions present in a given case. I don't think this a fair criticism, for the reason that there is not one instance where you see inflamed breasts that you do not have a certain class of remedies in your mind. This method we all pursue, and if this remedy did not suit the case we search for something new that suits the conditions. And, whatever they are, a certain class of remedies comes into our mind, and let us give those. That there are differential indications I do not deny; but let us help the younger man as he comes along. Dr. Biegler can say at a glance whether any of these remedies meet the case, but I see no objection at all to grouping remedies around the name of a disease, provided they are only prescribed upon special indications, and in these cases of much fever nine out of ten are met by Aconite, Belladonna, or Bryonia, and they will generally meet the conditions sure if they have not given Apis or something else.

Dr. Biegler—I called that up with the other points, not intending it for criticism. It is a question whether grouping a lot of remedies for certain conditions is not a mistake. It may lead into a ready method of selecting remedies. I only called it up for consideration.

Dr. Reed—If you have a case of diphtheria and the nurse says: "Doctor, we must be doing something." Are you not going to wash the throat or use a gargle? You must be doing something if you assent to a measure of that kind. You must also use local applications to the breast; and you are just as culpable in the one case as the other.

In a diseased condition of the nose or the throat, if you would in one place you must in another, and you must withstand the pressing urgency of the people. To resist this may seem foolish, but you had better leave the case than compromise your principles of homœopathics.

Dr. Sawyer—It seems to me that the course our Brother Custis recommends here is decidedly in the way of the beginner. He must examine his books before he prescribes or he will have a hard road to travel. He can look up the case with perfect safety without destroying the confidence of his patient—he can get along better in this way.

Dr. H. C. Allen—I usually advise patients to take, for instance, the mother-in-law and let her have the application on the breasts. She is able to stand it. It will not hurt her; she is well. If they insist on having a cataplasm or any other application in pneumonia that is all right, put it on the husband; he can stand it. This smoothes the thing over. But the best local application to any diseased condition of the mammæ is the same remedy which you give internally, potentized. If you are going to put anything there, locally, that is the thing to apply. Dr. Custis is certainly wrong in grouping his drugs. Repent, or you will be lost.

Dr. Guernsey—I would like to ask for the benefit of discussion whether anything can be offered in medication for retained placenta? If any members have had personal experience in such a case they might relate it.

Dr. Sawyer—I have had some experience, but I don't know that I can put it into sufficiently good shape to relate. I have stuck to the rule of giving the remedy covering the totality of the symptoms, and made the *Organon* do the work by the aid of medicine. In one case it was six weeks before all the placenta came away, but the patient made a splendid recovery. And in several cases where it was a day and a half before being expelled, I have seen no harm result where the conditions were met by the homœopathic remedy. I have never seen any septicæmia occur.

Dr. Nash—I once had a patient who was taken with profuse flowing, and I treated her from time to time without being able to more than just stop it. There was no apparent cause of hemorrhage. She was sure she had not miscarried. I had the impression forced upon me that there must be retained placenta, and she had miscarried without knowing it. The discharges became very offensive, and the woman was confined to bed three months with recurrent hemorrhages. The symptoms pointed to various remedies. I consulted Dr. Swan, who recommended Sabina$^{mm}$, which controlled it for a time, but it returned, and the discharge became very corrosive and of a horrible odor. I finally consulted Dr. Lippe, sending him a very careful history of the case. He sent me two powders of Sepia$^{cm}$, and I gave them, twenty-four hours apart. The first I gave at four P. M., and by seven severe pains came on with the expulsion of the partly disintegrated placenta. That was the end of the trouble.

Dr. Emory—If there are a few moments to spare I shall be very sorry to see this subject dropped. Is it the practice of other physicians under such a suspicion to make no efforts to ascertain the facts in the case? Dr. Bell, for instance, would not he use the dilator and curette? I have never had such a case, and I don't know what I would do under the circumstances.

Dr. Schmitt—I have had several experiences in that connection. I never had to resort to the curette. The indicated remedy brought away the placenta every time, even after a week's retention. One case, where I had tried different remedies but failed, I finally gave Lycopodium, the indicated remedy, in the morning at nine A. M., and the placenta passed at four A. M. the next day entirely, and the woman made a fine recovery. She had, in fact, the first symptoms of septicæmia. Another case was—

Dr. Reed—Was the retained placenta under your supervision?

Dr. Schmitt—Yes, she had a miscarriage—brought it on herself. I removed one placenta with the help of a placenta forceps formerly, and in the same patient afterward I got rid of the placenta by means of Lycopodium. In another case I attended a young married woman in her (two months) first pregnancy, and found her in terrible pains. The symptoms pointed to Sabina, and I gave one dose of the CM potency. In ten minutes I gave another dose, and she fell asleep. I waited an hour, but she did not wake. Next morning I got word to come up and see something. I then found that the placenta had passed during the night without a particle of pain.

Dr. Reed—Was she dead then?

Dr. Schmitt—No, she awoke next morning. I did not kill her.

Dr. Bell—I have had no experience in regard to Dr. Emory's question. I should have tried to remove the placenta by mechanical means, without too much interference; but I prefer a cure on homœopathic grounds. Such a cure is more satisfactory and more thorough. The merely mechanical removal is very difficult to make entire. There is no proper casting off of tissue; but in the other case there is a true casting off, and the tissues are left clean. If the case is going on to septicæmia, and there is great danger of death, then I feel we would be obliged to do the less regular thing, but I believe the other is better.

### X.—The Rochester Hahnemannian Hospital.

Dr. Biegler—It would be profitable for some one to make the announcement to this Association of the fact that a general Homœopathic Hospital and a general Hospital have been established in Rochester. The resolution comes rather late after the one just passed, but as our representative, who was appointed delegate to this Association, is not present, I take the liberty of calling your attention to this movement in Rochester, by way of a few words.

I think that we can now claim that this hospital is the first general Homœopathic Hospital that has ever been established, whose practice is based upon the teachings of the *Organon* strictly. The manner in which the hospital was established I will not relate in detail. It was under extraordinary difficulties. There is now a movement, with more influence, to establish a so-called Homœopathic Hospital, and it is nearly established, though not openly. That movement makes our undertaking a much more difficult one than under ordinary circumstances, and the resolution which has been offered, and any expression which will arise from that resolution, will be a source of considerable help. It is a question now of the right of one or other of these hospitals established in Rochester to exist. The place cannot support four hospitals. As there are now two larger ones in operation, and we have now started two new additional hospitals, and it is a struggle for the life of the one and the death of the other. For the purpose of maintaining influence and creating a public sentiment against us, the State Society lends its influence by going to Rochester this summer to hold its meeting for the purpose of influencing the project on that side. Therefore, the resolution offered by Dr. Bell will not only be gratefully received by the representatives of this Association and Homœopathy in general, but it seems to me a necessity, in order to give us moral support, that the people may see we are not standing alone. The movement which has been inaugurated in Rochester has had the effect of making many people understand. Many now know what Homœopathy is, and what the practice of pure Homœopathy is. A large class are dazed and confused, and a portion of that class are not aware but—what I believe—that we are an offshoot from the regular homœopathic school, so-called, that we have gone off on some strange notions, separated from the regular body of the homœopathic school, and established something different, and I, for one, will welcome any expression you may give us for that reason.

