# Erratum

**Published:** 1889-12

**Authors:** 


ERRATUM.—In our September issue, page 389, line 19th from bottom, for *Organon* read *Organism.*

